# Hypericin-photodynamic therapy induces human umbilical vein endothelial cell apoptosis

**DOI:** 10.1038/srep18398

**Published:** 2015-12-17

**Authors:** Qian Zhang, Zhuo-heng Li, Yuan-yuan Li, San-jun Shi, Shi-wen Zhou, Yuan-yuan Fu, Qing Zhang, Xue Yang, Ruo-qiu Fu, Lai-chun Lu

**Affiliations:** 1 Department of Pharmacy, Institute of Surgery Research, Daping Hospital, Third Military Medical University, Chongqing, 400042, P. R. China; 2 Office of Clinical Pharmacological Center, Xinqiao Hospital, Third Military Medical University, Chongqing, 400037, P. R. China

## Abstract

The conventional photosensitizers used in photodynamic therapy (PDT), such as haematoporphyrin (HP), have not yet reached satisfactory therapeutic effects on port-wine stains (PWSs), due largely to the long-term dark toxicity. Previously we have showed that hypericin exhibited potent photocytotoxic effects on Roman chicken cockscomb model of PWSs. However, the molecular mechanism of hypericin-mediated photocytotoxicity remains unclear. In this study, we employed human umbilical vein endothelial cells (HUVECs) to investigate the hypericin-photolytic mechanism. Our study showed that hypericin-PDT induced reactive oxygen species (ROS), resulting in cell killings and an activation of the inflammatory response. Importantly, we have also discovered that photoactivated hypericin induced apoptosis by activating the mitochondrial caspase pathway and inhibiting the activation of the vascular endothelial growth factor-A (VEGF-A)-mediated PI3K/Akt pathway. Notably, we found that hypericin exhibited a more potent photocytotoxic effect than HP, and largely addressed the inconvenience issue associated with the use of HP. Thereby, hypericin may be a better alternative to HP in treating PWSs.

Port-wine stains (PWSs) are a congenital form of vascular malformation that are characterized by ectatic capillaries and postcapillary venules in the papillary and mid-reticular layers of the dermis, often occurring on the face and neck. PWSs are the most common type of vascular malformation, with a prevalence of approximately 0.3% in newborns^1^,^2^. Over time, the diameters of the vessels would progress, resulting in clinical condition and even a disfiguring lesion^3^. It is estimated that two-thirds of patients would develop hypertrophic or nodular lesions by the age of fifty^4^,^5^,^6^.

Recently, photodynamic therapy (PDT) has been successfully used to treat PWS lesions^7^,^8^. The process involves the use of a photosensitizer, light and molecular oxygen. Nonetheless, the photosensitizer is a critical factor in effective treatment, which reacts with molecular oxygen to produce cytotoxic reactive oxygen species (ROS) to destroy the diseased tissue^9^. Current studies have focused on identifying satisfactory photosensitizer drugs, such as haematoporphyrin and its derivatives. However, the half-life of haematoporphyrin and its derivatives are very long and the fluorescence can still be detected even after a year following the intravenous infusion^10^,^11^. This long half-life can lead to many complications, such as dark toxicity, due to the prolonged skin photosensitivity to sunshine and visible light. Consequently, when administered with haematoporphyrin or their derivatives, patients are required to avoid sun exposure for up to 4 weeks^12^,^13^. This poor patient compliance has adversely limited their clinic applications^14^.

Hypericin is a naturally occurring photosensitizer extracted from a plant of *Hypericum perforatum* (St. John’s Wort)^15^. It exhibits high ROS yields (0.17–0.80)^16^,^17^. Studies have shown that hypericin is a selective anti-tumour photosensitizer^17^. And many studies have revealed that its anti-tumour properties are mediated by autophagy and immune responses^18^,^19^,^20^,^21^,^22^. However, a few studies have been conducted which indicated that treating effects of hypericin on vascular injury and the elimination of malformed capillaries^23^. For example, our previous investigations first revealed that hypericin was used in treatment of PWSs using a chicken comb model, and good therapeutic effect was obtained^24^. This result is very exciting, especially when considering that the half-lives of hypericin is only 27 h even at a dosage of 1500 μg/kg^25^. The much shorter half-life of hypericin has therefore largely addressed the inconvenience issue associated with the use of other photosensitizers. The great potential of hypericin as an excellent alternative photosensitizer therefore prompted us to further explore its reaction mechanism.

In this study, human umbilical vein endothelial cells (HUVECs) were used as the model to investigate how hypericin induces photocytotoxicity. We found that hypericin induced apoptosis in HUVECs. Specifically, we evaluated the effects of hypericin on the mitochondrial apoptotic pathway and the VEGF-A-mediated PI3K/Akt pathway. We discovered that hypericin induced apoptosis *via* Bax/Bcl-2 ratio upregulation, ΔΨ_m_ collapse, accompanied with cyto c release, procaspase-3, procaspase-9 and PARP cleavage, and also VEGF-A suppression, p-Akt inhibition and Bad dephosphorylation. The efficacy of hypericin was also compared to haematoporphyrin. Hypericin exhibited a more potent photocytotoxic effect on the above-mentioned pathways than haematoporphyrin. These findings provide a novel mechanistic basis for the application of hypericin in the treatment of PWSs.

## Materials and Methods

### Compounds

Hypericin (HY) was purchased from Chengdu Biopurify Phytochemicals (batch no. 081204, purity 99.1%, Chengdu, China). HY was dissolved in dimethylsulfoxide (DMSO, Sigma-Aldrich Co., St Louis, MO) to produce a stock solution (3.2 mM) for the *in vitro* assays. Haematoporphyrin (HP) was obtained from Shanghai Red and Green Photosensitizer Institute (Shanghai, China) and dissolved in DMSO to produce a stock solution (1.6 mM) for the *in vitro* assays. The HY and HP stock solutions were stored at −20 °C and diluted to the final concentration using complete Roswell Park Memorial Institute 1640 (RPMI-1640, New Mexico, USA) medium before use. zVAD-fmk was obtained from Selleck Chemicals (Houston, USA) and Bax-Inhibiting Peptide (BIP) was purchased from Merck Millipore (Hesse-Darmstadt, GER). VEGF-A (vascular endothelial growth factor-A) was obtained from Cell Signaling Technology (CST, Boston, USA).

### Cell lines and cell culture

Human umbilical vein endothelial cells (HUVECs) were obtained from American Type Culture Collection (Rockefeller, Maryland, USA). The cells were incubated in complete RPMI-1640 medium supplemented with 10% (v/v) heat-inactivated foetal bovine serum, 2 mM L-glutamine and a penicillin-streptomycin solution (100 U/ml penicillin and 100 μg/ml streptomycin) at 37 °C with 5% CO_2_ and 95% air in a humidified atmosphere.

### *In vitro* PDT treatment

PDT was performed on HUVECs seeded into cell culture plate. After an overnight incubation, the media were replaced with fresh media containing increasing concentrations of HY or HP. The cells were then incubated for 24 h in the dark. After replacing the media, the cells were then irradiated using a 585-nm LED light (YK-LED-PDT; Jiangxi Xinyu Shunxing Technology Development Co., Jiangxi, China) at a dose of 1.0 J/cm^2^. The HUVECs treated in the same way in the absence of the PS drugs (HY or HP) were used as the vehicle control.

### Inhibitor treatment

The HUVECs were pretreated with the pan-caspase inhibitor zVAD-fmk (20 μM) or the more specific Bax inhibitor BIP (100 μM) for 3 h followed by treatment with HY (0.062 μM) in combination with the inhibitor for 24 h. Then the cells PDT-treated were cultured for 24 h. The HUVECs treated in the same way in the absence of HY were used as the zVAD-fmk/BIP control.

### VEGF stimulation

The HUVECs were serum-starved for 24 h and stimulated with VEGF-A (25 ng/ml) in fresh medium. The cells were then incubated with media containing HY (0.062 μM) and VEGF-A (25 ng/ml) for 24 h; the PDT-treated cells were cultured for an additional 24 h. The cells were lysed and the proteins were harvested for western blot analysis.

### Observation of the morphological changes

The HUVECs were seeded onto a 6-well plate (1.5 × 10^5^ cells per well). The HUVECs treated in a similar manner but without light irradiation (i.e., PSs treatment without light irradiation (Non-PDT)) were used as controls. The changes in the celluar morphology were observed using an inverted microscope (Olympus, TAKACHIHO SEISAKUSHO, Japan).

### Cell viability assays

MTT assays were conducted to determine the photocytotoxic effects of HY on HUVECs. The cells were divided into two groups (PDT or Non-PDT group). Approximately 5.0 × 10^3^ cells were seeded onto 96-well plates. After an overnight growth, the cells were treated with increasing concentrations of HY (0.016, 0.031, 0.062, 0.125, 0.250, and 0.500 μM) and HP (2 μM) for 24 h. The culture media were removed and replaced with fresh media. One group of cells (PDT group) was then exposed to a 585-nm LED light at a dose of 1.0 J/cm^2^. The other group (Non-PDT group) was not treated with light irradiation. Afterwards, both groups were incubated for an additional 24 h in the dark. Then, 5 mg/ml MTT (3-[4,5-dimethylthiazol-2-yl]-2,5-diphenyltetrazolium bromide, Sigma-Aldrich Co., St Louis, MO) was added to each well, and the cells were incubated for another 4 h as previously described. The cell viability of the treated cells was compared with that of the vehicle-only control cells (100%) to calculate the IC_50_ value.

### *DAPI* staining assay

The HUVECs were seeded onto a 6-well plate (2 × 10^5^ cells per well) before the PDT treatment. After treatment, the media were aspirated and the cells were fixed with 4% paraformaldehyde for 5 min. The fixed cells were then washed twice with phosphate-buffered saline (PBS) and stained with *DAPI* (4′,6-diamidino-2-phenylindole, Beyotime, Shanghai, China) for 5 min at room temperature. After three washes with PBS times, the condensed chromatin and fragmented nuclei in *DAPI*-stained apoptotic cells were viewed under a fluorescence microscope (Nikon Co., Japan; magnification: ×400).

### Flow cytometry

The HUVECs were seeded onto a 6-well plate (2 × 10^5^ cells per well). The irradiated cells were then harvested after the PDT treatment. The early apoptotic cells (Annexin V-FITC^+^/PI^−^), late apoptotic cells and dead cells (Annexin V-FITC^+^/PI^+^) were quantitatively estimated using a FACScan flow cytometer (FACSVantage SE, Becton-Dickinson, New Jersey, USA) using the Annexin V-FITC and PI apoptosis detection kit (Beyotime, Shanghai, China).

### Quantification of the secreted TNF-α, IL-6, IFN-γ, IL-1β and IL-10 levels using ELISAs

The HUVECs were seeded onto a 24-well plate (2.0 × 10^4^ per well) before the PDT treatment. The cell culture supernatants were then collected and filtered through a 0.22-μm Steriflip filter (Millipore, Massachusetts, USA). The concentrations of TNF-α, IL-6, IFN-γ, IL-1β and IL-10 were quantified using the Human ELISA Kit (BOSTER, Wuhan, China) following the manufacturer’s instructions.

### Measurement of the intracellular ROS levels

The ROS levels were measured using the Reactive Oxygen Species Assay Kit (Beyotime, Shanghai, China) following the manufacturer’s instructions. Intracellular ROS production was assessed by measuring the oxidized dichlorofluorescein (DCF) levels in the HUVECs. The non-fluorescent probe 2′,7′-dichlorofluorescein diacetate (DCFH-DA) diffuses into the cells, where it is deacetylated to non-fluorescent 2′,7′-dichlorofluorescein (DCFH). Subsequently, DCFH is oxidized by the intracellular ROS to the fluorescent product DCF. The cells were seeded onto a 6-well plate (2 × 10^5^ cells per well) before the PDT treatment. The irradiated cells were then harvested and incubated with DCFH-DA (25 μM) for 45 min at 37 °C in the dark. Afterwards, the DCFH-DA was removed and the cells were washed three times with PBS. The fluorescence was read at 485 nm (excitation) and 535 nm (emission) with a fluorescence spectrophotometer (Hitachi, Ltd, Japan).

### Mitochondrial membrane potential (ΔΨm) assay

The ΔΨm was measured using the mitochondrial membrane potential assay kit with JC-1 (5,5′,6,6′-tetrachloro-1,1′,3,3-tetraethylbenzimidazole-carbocyanide iodine, Beyotime, Shanghai, China) following the manufacturer’s instructions, using a fluorescence microscope (Nikon Co., Japan; magnification x400). The HUVECs were seeded onto a 6-well plate (2 × 10^5^ cells per well) before the PDT treatment. Afterwards, the cultured cells were stained with JC-1 (10 μg/ml) for 30 min at 37 °C and washed twice with ice-cold PBS. The cells incubated with carbonyl cyanide m-chlorophenylhydrazone (protonophore, CCCP, 10 μM) were used as the positive control because CCCP effectively causes ΔΨ_m_ depletion. The excitation and emission wavelengths were 510 nm and 527 nm, respectively, for the detection of the monomeric form of JC-1 (green fluorescence). The excitation and emission wavelengths of 580 nm and 590 nm, respectively, were used to detect JC-1 aggregation (red fluorescence). The ratio of red (i.e., the aggregated form of JC-1) to green (i.e., the monomeric form of JC-1) fluorescence intensity, which represented the ΔΨm of HUVECs, was normalized to that of the vehicle control (100%). The fluorescence intensity was evaluated using the ImageJ 2 × software (National Institutes of Health, USA).

### Western blot analysis

The cells were grown on a 6-well plate (2 × 10^5^ cells per well) before the PDT treatment. After treatment, the media were aspirated and the cells were washed twice with ice-cold PBS. The proteins were harvested from the cells using cell lysis buffer containing 1 mM PMSF (Beyotime, Shanghai, China). The protein concentrations were measured using the BCA Protein Assay Kit (Beyotime, Shanghai, China).

The proteins were separated by 10% or 12% (for Bcl-2, Bax, cleaved caspase-9, procaspase-3, cleaved PARP, VEGF-A, phospho-Akt (Ser473, p-Akt), Akt, Bad and β-Actin) SDS-polyacrylamide gels and electrophoretically transferred onto PVDF membranes (Millipore, Massachusetts, USA). The membranes were blocked with 6% non-fat milk at 37 °C for 1 h and then incubated overnight with the corresponding primary antibodies at 4 °C. The following antibodies were used in this study: anti-Bcl-2 (1:500, CST, Boston, USA), anti-Bax (1:500, CST, Boston, USA), monoclonal anti-cleaved caspase-9 (1:1000, CST, Boston, USA), monoclonal anti-procaspase-3 (1:5000; Abcam, Cambridge Science Park, UK), anti-cleaved PARP (1:500; CST, USA), polyclonal anti-VEGF-A (1:500, Abcam, Cambridge Science Park, UK), monoclonal anti-p-Akt (Ser473,1:500, CST, USA), anti-Akt (1:500, CST, USA), polyclonal anti-Bad (1:1000, CST, Boston, USA) and anti-β-Actin (1:1000, CST, Boston, USA). The blots were incubated with peroxidase-conjugated AffiniPure goat anti-rabbit or anti-mouse IgG (1:5000, ZSGB-BIO, Beijing, China) as the secondary antibodies for 1 h at 37 °C. The blots were detected with the Immobilon Western Chemiluminescent HRP Substrate (Millipore, Massachusetts, USA). The protein levels were quantitated by densitometry using AlphaEaseFC 4.0 software (San Leandro, CA) and then normalized to the vehicle control (100%). β-Actin was used as an internal control.

### qRT-PCR

The HUVECs were grown on a 6-well plate (2 × 10^5^ cells per well) before the PDT treatment. After treatment, the cells were then washed twice with ice-cold PBS. Total RNA was extracted from the PDT-treated cells using an RNAiso Plus Kit (TaKaRa, Tokyo, Japan) following the manufacturer’s instructions. Reverse transcription of the extracted RNA was performed using the PrimeScriptTM RT Reagent Kit with gDNA Eraser (TaKaRa, Tokyo, Japan). Two-step qRT-PCR reactions were performed using SYBR® Premix Ex TaqTM II (TaKaRa, Tokyo, Japan) on a Real-Time qPCR System (Mx3000P, Agilent Stratagene, USA). The primers used in this study are listed in Table 1. The relative fold change in the mRNA expression of the target gene was quantified using the 2^−∆∆CT^ method and the MXProv4.1 software. Glyceraldehyde 3-phosphate dehydrogenase (GAPDH) was used as a housekeeping gene. A vehicle-only control was used as a calibrator for quantification.

### Statistical analysis

The data were expressed as means plus or minus standard deviation (means ± S.D.). Statistical analysis was performed by one-way analysis of variance (ANOVA). *P* < 0.05 was regarded as a statistically significant difference.

## Results

### Growth inhibition by HY-PDT

As shown in Supplementary Fig. S1, after 24 h the growth of HUVECs treated with PDT treatment were significantly inhibited and down-regulated procaspase-3 and up-regulated cleaved PARP were observed. Thus, the subsequent experiments were implemented exposed to photosensitizer dugs with PDT treatment after 24 h.

**Figure 1:**
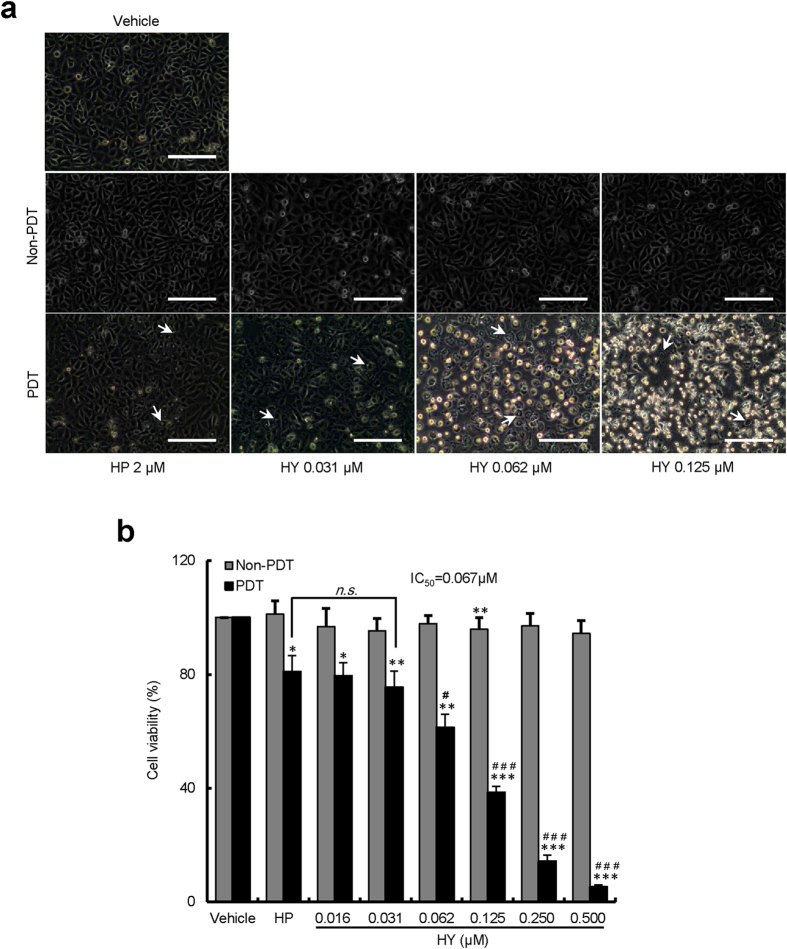
Inhibition of the growth of HUVECs by HY-PDT. The HUVECs were divided into two groups (with or without PDT treatment). The cells were treated with increasing concentrations of HY (0.016–0.500 μM) or HP (2 μM). One group of cells (PDT group) was then exposed to a 585-nm LED light at a dose of 1.0 J/cm^2^. The other group (Non-PDT group) was treated without light irradiation. All the cells were incubated for an additional 24 h. (**a**) The effects of HY- or HP-PDT on morphological changes in HUVECs. The images are representative of three independent experiments. The white arrows indicate the apoptotic cells. Bars, 100 μm. (**b**) The inhibitory effect of HY- or HP-PDT on HUVEC growth was analysed using the MTT assay. The viability of the treated cells was compared to the vehicle-only control cells (100%). Data are presented as means ± S.D. (n = 3); *P < 0.05, **P < 0.01, ***P < 0.001, compared to vehicle control; ^#^*P* < 0.05, ^###^*P* < 0.001, compared to HP, *n.s.*, *P* > 0.05.

To determine whether increasing the concentrations of hypericin (HY) could inhibit the growth of HUVECs, we perform image studies using an inverted microscope, given that cell morphology is an indicator of health in cell culture. During the experiment, we observed a significant morphological change in HUVECs following the HY-PDT. As shown in Fig. 1a, the HUVECs treated with photosensitizer drugs without light irradiation (Non-PDT) had a cobble stone-like shape, and there was no sign of cell apoptosis. In contrast, after PDT treatment with 2 μM haematoporphyrin (HP) and 0.031 μM HY, blebbing was observed on the cell membrane, which is a sign of early apoptosis. Moreover, as the concentration of HY (0.062 and 0.125 μM) was increased, more cells became rounded, shrunken and detached from each other, some floated in the medium. These are prominent morphological signs of apoptosis.

To further study the effect of photosensitizer-PDT on cell viability, the HUVECs were treated with HY or HP-PDT and after 24 h were subjected to the MTT assay. As shown in Fig. 1b, the HUVECs’ viability decreased from 79.5% to 5.2% in the irradiated cells treated with HY concentrations ranging from 0.016 to 0.5 μM (*P* < 0.05 vs. vehicle control). Statistical analysis indicated that the IC_50_ value was 0.067 μM. However, the viability of the 2 μM HP-PDT was 81.0%. No significant differences were found between 0.031 μM HY-PDT and 2 μM HP-PDT (*P* > 0.05). The inhibitory effect with increasing concentrations of HY (HY 0.062-0.5 μM) was stronger than that with 2 μM HP. This is very exciting, the low dosage may allow for low toxicity. However, the cell viability was not inhibited in Non-PDT cells (*P* > 0.05 vs. vehicle control), demonstrating a possible selective treating using HY.

### HY-PDT induced apoptosis in HUVECs

**Figure 2:**
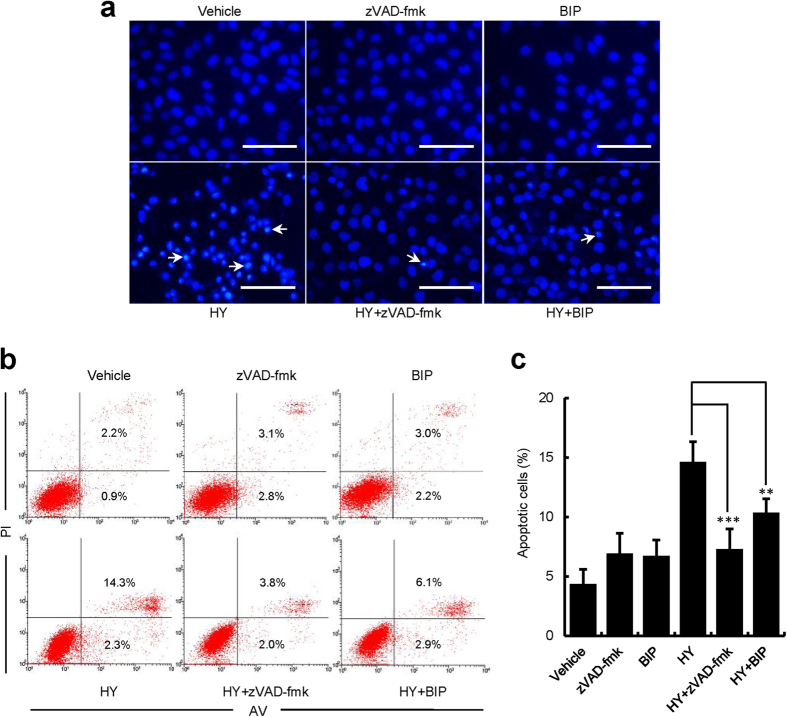
HY-PDT-induced HUVEC apoptosis. The HUVECs were pretreated with pan-caspase inhibitor zVAD-fmk (20 μM), or more specific Bax inhibitor Bax-Inhibiting Peptide (BIP) (100 μM) for 3 h, followed by treatment with HY (0.062 μM) in combination with the inhibitor for 24 h. The cells were irradiated with a 585-nm LED light at a dose of 1.0 J/cm^2^. The cells were cultured for an additional 24 h and were harvested for analysis. (**a**) The HUVECs were stained with DAPI to label the cell nuclei and observed by fluorescence microscopy. The images are representative of three independent experiments. The white arrows indicate the condensed chromatin, marginalization or nuclei fragmented into apoptotic bodies. Bars, 100 μm. (**b,c**) Flow cytometry analysis of the apoptotic cells. The bar graphs show the percentage of apoptotic cells for each of the indicated conditions. Data are presented as means ± S.D. (n = 3), **P < 0.01, ***P < 0.001.

To investigate whether the HY-PDT-mediated inhibition of cell growth result from the induction of apoptosis, we examined the characteristics changes in chromatin morphology, which is an indicative sign of apoptosis. The vehicle control cells exhibited homogenous chromatin morphology. After the treatment using HP-PDT and HY-PDT, the cells exhibited the morphological features of apoptotic cells, such as chromatin condensation, marginalization, or nuclear fragmentation (Supplementary Fig. S2a). Specifically, the DNA fragmentation was observed at high concentration of HY (0.125 μM). However, this apoptosis was markedly decreased when pan-caspase inhibitor zVAD-fmk (20 μM), or a more specific Bax inhibitor BIP (100 μM) was added (Fig. 2a). This study suggested that HY-PDT inhibited the growth of HUVECs *via* induction of apoptosis.

To further confirm the process of HY-PDT-induced apoptosis, flow cytometry was applied to identify the apoptotic cells (Annexin V-FITC^+^/PI^−^) and the late apoptotic or dead cells (Annexin V-FITC^+^/PI^+^). As shown in Supplementary Fig. S2b, the percentages of apoptotic cells increased to 6.0%, 6.3%, 17.8%, and 48.4% upon the treatment of 0.031, 0.062, 0.125 μM HY and 2 μM HP, respectively, indicating that HY-PDT led to a concentration-dependent increase in the percentage of apoptotic cells. In addition, the number of apoptotic cells increased even further after treatment with higher concentrations of HY (0.062 and 0.125 μM) than with HP (2 μM) (Supplementary Fig. S2c). We found that the percentage of apoptotic cells was strongly reduced in the HUVECs pretreated with z-VAD-fmk or BIP (Fig. 2b,c). Taken together, our studies confirm that HY-PDT induced HUVEC apoptosis, the effect of which displayed a more potent on the inhibition of cell growth *via* apoptosis.

### HY-PDT promoted the inflammatory response in HUVECs

**Figure 3:**
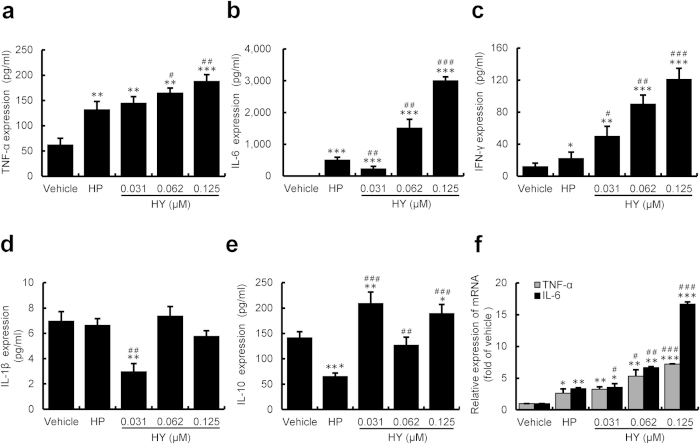
HY-PDT-promoted secretion of inflammatory mediators in HUVECs. The cells were incubated with increasing concentrations of HY (0.031, 0.062, 0.125 μM) or HP (2 μM) for 24 h followed by PDT for 24 h post-incubation. The secreted inflammatory mediators were measured in the cell culture supernatants. The secretion of TNF-α (**a**), IL-6 (**b**), IFN-γ (**c**), IL-1β (**d**) and IL-10 (**e**) were measured by ELISAs. The data are presented as the means ± S.D. of three independent experiments; *P < 0.05, **P < 0.01, ***P < 0.001, compared to the vehicle control; ^#^*P* < 0.05, ^##^*P* < 0.01, ^###^*P* < 0.001, compared to HP. (**f**) Effect of HY- or HP-PDT on TNF-α and IL-6 mRNA expression, as examined by qRT-PCR. The gene expression was normalized to glyceraldehyde-3-phosphate dehydrogenase (GAPDH) and calibrated to the vehicle control. Data are presented as means ± S.D. (n = 3); *P < 0.05, **P < 0.01, ***P < 0.001, compared to the vehicle control; ^#^*P* < 0.05, ^##^*P* < 0.01, ^###^*P* < 0.001, compared to HP.

To test the level of inflammatory mediators in HUVECs following PDT, the expression of TNF-α, IL-6 and IFN-γ was determined using ELISAs and qRT-PCR assays. Our studies showed that HY- or HP-PDT strongly enhanced the secretion of TNF-α, IL-6 and IFN-γ (Fig. 3a–c). Similarly, the expression of the TNF-α and IL-6 mRNAs also concentration-dependently and significantly increased, as shown in Fig. 3f. Again, in this case, HY exhibited a more potent effect than 2 μM HP did. Notably, the IL-6 levels were clearly increased compared to the TNF-α and IFN-γ levels. However, the IL-1β and IL-10 levels were inconsistent after PDT and the concentration of IL-1β mostly remained below the detection limit (Fig. 3d,e). These results demonstrate that HY- or HP-PDT treatment promoted the inflammatory response, resulting in the secretion of TNF-α, IL-6, IFN-γ, particularly IL-6, which consequently led to HUVEC death. Additionally, our studies also indicated that HY-PDT exhibited a much more potent effect than HP-PDT in terms of activating the immune response.

### HY-PDT enhanced ROS production in HUVECs

**Figure 4:**
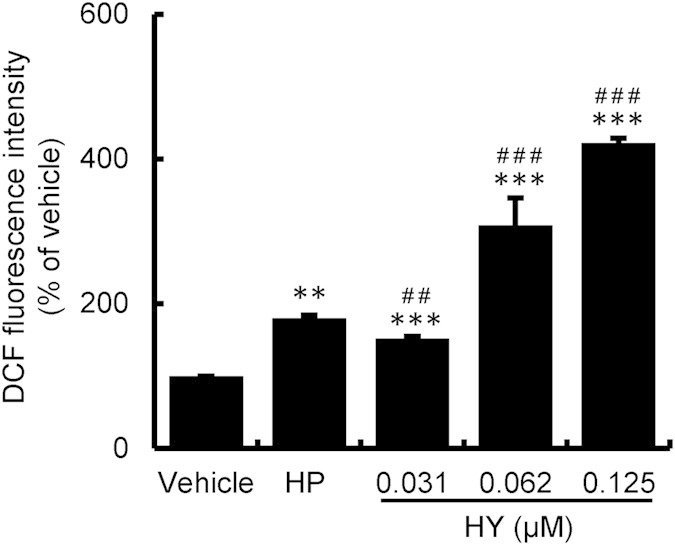
HY-PDT-triggered ROS induction in HUVECs. The HUVECs were treated with increasing concentrations of HY (0.031, 0.062, 0.125 μM) or HP (2 μM) for 24 h and were then exposed to a 585-nm LED light at a dose of 1.0 J/cm^2^. The cells were then incubated for 24 h. Intracellular ROS production was assessed by measuring the oxidized DCF levels in HUVECs. Data are presented as means ± S.D. (n = 3); **P < 0.01, ***P < 0.001, compared to the vehicle control; ^##^*P* < 0.01, ^###^*P* < 0.001, compared to HP.

Our next goal was to study whether ROS production contributed to the secretions of inflammatory mediators. The ROS production was measured using a fluorescent probe DCFH-DA. As shown in Fig. 4, the level of DCF fluorescence was significantly increased in the HUVECs treated with HY-PDT in a concentration-dependent manner. At higher concentrations (0.062 and 0.125 μM), the fluorescence intensities from HY-PDT were greater than that from 2 μM HP-PDT. These observations indicated that the oxidized DCF levels were high in HUVECs, indicating a high level of ROS production following the HY- or HP-PDT treatment. Taken together, our studies suggest that HY-PDT triggered ROS production, which was more effective than that of HP-PDT.

### HY-PDT led to collapse of the ΔΨ_m_ in HUVECs

**Figure 5:**
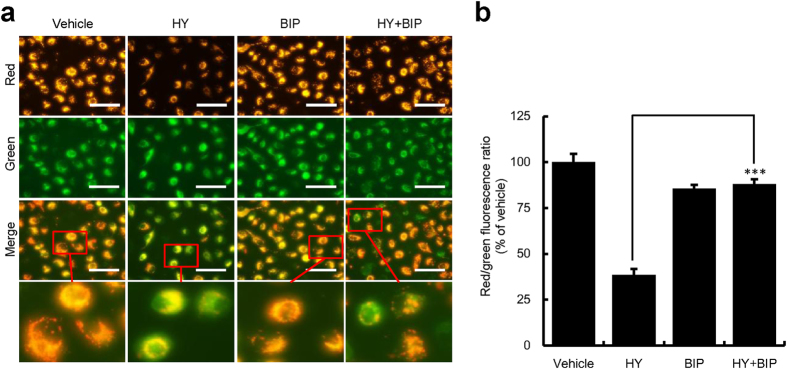
HY-PDT-induced ΔΨ_m_ collapse in HUVECs. The HUVECs were pre-exposed to the specific Bax inhibitor BIP (100 μM) for 3 h before exposure to HY (0.062 μM) for 24 h. The cells were irradiated using a 585-nm LED light at a dose of 1.0 J/cm^2^ and were incubated for 24 h. (**a**) The images were captured using fluorescence microscopy and are representative of three independent experiments. Bars, 100 μm. (**b**) The bar graphs show the ratios of red and green fluorescence intensities from JC-1, as detected by fluorescence microscopy. The ratios of the red and the green fluorescence intensities were normalized to that of the vehicle control (100%). Data are presented as means ± S.D. (n = 3), ***P < 0.001.

Apoptosis is activated through two major pathways, mitochondrion dependent or independent pathway. The ΔΨm is an indicator of mitochondrial function. To investigate the mechanism of apoptosis, the ΔΨm was examined by fluorescence microscopy. The ratio of red and green fluorescence intensities represented the ΔΨm of HUVECs. CCCP can cause ΔΨm depletion and was, therefore, used as a positive control. As shown in Supplementary Fig. S5a, following PDT with increasing concentrations of HY, the red fluorescence became weaker and the green fluorescence became stronger. As shown in Supplementary Fig. S5b, the ratio of red and green fluorescence intensities decreased significantly upon the treatment of HY-PDT; and the rate of decrease is concentration-dependent, indicating that HY is a potent photosensitizer that leads to collapse of the ΔΨm. Compared with the CCCP control, the ratios of HP and HY at higher concentrations (0.062 and 0.125 μM) were not significantly different (data not shown), indicating that HY- or HP-PDT, could both cause the loss of ΔΨm in HUVECs. However, the specific Bax inhibitor BIP (100 μM) could significantly relieve the HY-induced (0.062 μM) mitochondrial depolarization, as shown in the fluorescent colour change from green to orange-red. This study demonstrated an attenuation of the dissipation of ΔΨm (Fig. 5a,b). Mitochondrial dysfunction has been suggested to be an important factor in the process of apoptosis. Collectively, these results showed that HY-PDT could lead to a significant collapse of ΔΨm and subsequently apoptosis. These findings therefore, indicated that HY can induce apoptosis *via* a mitochondria-dependent pathway.

### Apoptosis was induced *via* the mitochondrial pathway of caspase activation in HUVECs

**Figure 6:**
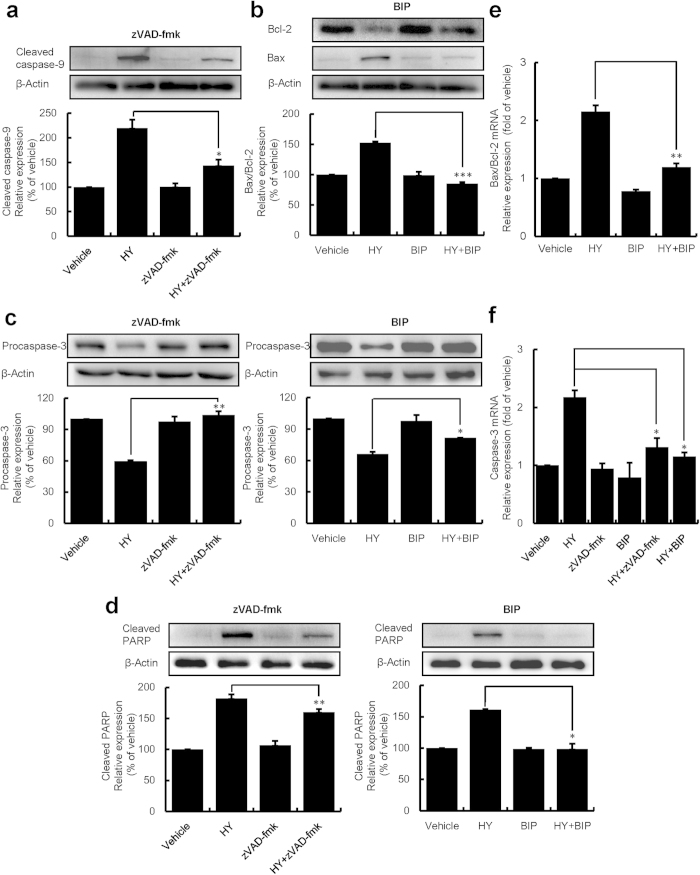
HY-PDT-induced expression of apoptotic mediators in HUVECs. The HUVECs were pretreated with either the pan-caspase inhibitor zVAD-fmk (20 μM) or the more specific Bax inhibitor BIP (100 μM) for 3 h followed by treatment with HY (0.062 μM) for 24 h. The cells were then exposed to a 585-nm LED light at a dose of 1.0 J/cm^2^. The cells were incubated for an additional 24 h. The total proteins and RNA were extracted from the PDT-treated cells. Western blot analysis and two-step qRT-PCR reactions were performed, respectively. Western blot analysis of cleaved caspase-9 (**a**), Bcl-2 and Bax (**b**), procaspase-3 (**c**) and cleaved PARP (**d**) in HUVECs treated with PDT. Densitometric measurements were analysed using AlphaEaseFC 4.0 software. The protein expression levels were normalized to those of vehicle control (100%). Data are presented as means ± S.D. (n = 3); *P < 0.05, **P < 0.01, ***P < 0.001. The Bax/Bcl-2 (**e**) and caspase-3 (**f**) mRNA levels were assessed by qRT-PCR. The gene expression levels were normalized to those of GAPDH and were calibrated to vehicle controls. Data are presented as the means ± S.D. (n = 3); *P < 0.05, **P < 0.01.

To further explore the mechanism underlying the apoptosis, western blot analyses of apoptosis related proteins and qRT-PCR analyses of mRNAs were performed (Fig. 6 and Supplementary Fig. S6). Concentration-dependent down-regulation in the expression of the anti-apoptotic protein Bcl-2 and up-regulation of the pro-apoptotic protein Bax were found in HUVECs following HY- or HP-PDT (Supplementary Fig. S6a). A similar trend in the expression of procaspase-3 and cleaved PARP was observed (Supplementary Fig. S6a,c). An increase of the cleaved caspase-9 protein was also detected in the HY- or HP-treated cells (Fig. S6a,b). These effects were further studied by measuring of the genes involved in apoptosis using qRT-PCR. Our studies revealed that HY- or HP-PDT significantly increased the Bax/Bcl-2 ratio, cyto c and caspase-3 mRNAs (Supplementary Fig. S6d–f). In this study, we have also found that HY-PDT at high concentrations (0.062 and 0.125 μM) was more effective than the 2 μM HP-PDT.

Additionally, we found that both zVAD-fmk and BIP attenuated the HY-induced (0.062 μM) effects on the levels of the Bax/Bcl-2, cleaved caspase-9, proacaspase-3 and cleaved PARP proteins (Fig. 6a–d). Similarly, the mRNA up-regulations of the Bax/Bcl-2 and caspase-3 also decreased by zVAD-fmk or BIP (Fig. 6d,e). These results further confirmed that HY- or HP-PDT induced apoptosis through the mitochondrial caspase pathway, which was initiated by an increase in the Bax/Bcl-2 ratio, resulting in a loss of ΔΨm (Fig. 5), leading to simultaneous release of cyto c to further activate caspases (caspase-9 and caspase-3), and eventually resulting in PARP cleavage.

### HY-PDT blocked the PI3K/Akt (phosphatidylinositol 3-kinase/protein kinase B) pathway

**Figure 7:**
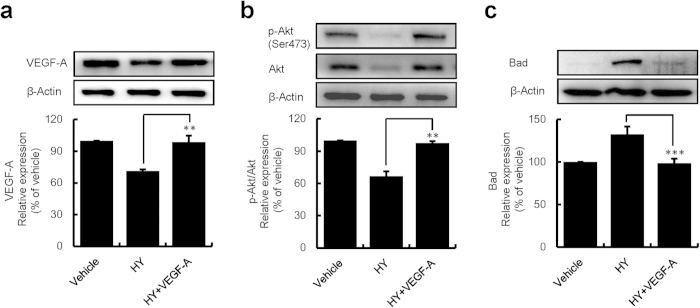
Inhibition of VEGF-A-mediated PI3K/Akt pathway in HUVECs. The HUVECs were serum-starved for 24 h and stimulated in fresh medium with VEGF-A (25 ng/ml) and then treated with HY (0.062 μM) in combination with VEGF-A (25 ng/ml) for 24 h. The cells were exposed to a 585-nm LED light at a dose of 1.0 J/cm^2^ and were incubation for 24 h. The cells were lysed and the proteins were harvested for western blot analysis of VEGF-A (**a**), p-Akt (Ser473) and Akt (**b**), and Bad (**c**). Densitometric measurements were analysed using AlphaEaseFC 4.0 software. The protein expression levels were normalized to those of the vehicle control (100%). Data are presented as means ± S.D. (n = 3); **P < 0.01, ***P < 0.001.

The PI3K/Akt signalling pathway has been shown to be a vital survival signalling pathway and is induced by VEGF-A^26^. Studies have shown that VEGF suppression can inhibit carcinoma cell proliferation^27^,^28^. To investigate whether HY-PDT can interfere the PI3K/Akt pathway, the expression of VEGF-A, p-Akt, and total Akt proteins, the expression of protein and mRNA for the downstream (effector), as well as Bad, were measured (Fig. 7 and Supplementary Fig. S7). Compared to the vehicle control, the expression of the VEGF-A, total Akt and p-Akt (p-Akt/Akt ratio) proteins all decreased in concentration-dependent manner (Supplementary Fig. S7a–c). We also found that the value of Bad increased in a concentration-dependent manner at the transcriptional level and the translational level (Supplementary Fig. S7a,d,e). Moreover, HY-PDT (0.062 and 0.125 μM) was more effective against the above-mentioned effectors compared to 2 μM HP (Supplementary Fig. S7).

Similarly, as shown in Fig. 7, VEGF-A (25 ng/ml) significantly attenuated the HY-PDT-induced (0.062 μM) decrease in the expression of the VEGF-A, the total Akt and p-Akt (p-Akt/Akt ratio) proteins and Bad activation (Fig. 7a–c). These results suggested that HY- and HP-PDT were able to suppress VEGF-A expression, thereby inhibiting Akt activation, and consequently leading to the increase in Bad expression. These studies supported the hypothesis that HY-PDT inhibited the VEGF-A-mediated PI3K/Akt pathway, which involves alternative mechanisms to inhibit cell growth.

### HY-PDT induced cell death in HUVECs

**Figure 8:**
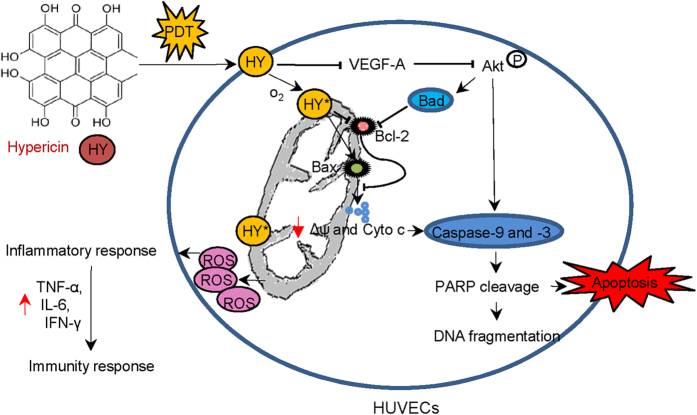
HY-PDT-induced cell death in HUVECs. HY is activated by yellow light at 585 nm in the presence of molecular oxygen to generate cytotoxic ROS, thereby inducing cell death, activating the inflammatory responses, and leading to apoptosis.

The above results suggested that cell death mechanisms underlie the role of HY-PDT in HUVECs. HY, a lipophilic polycyclic quinine, preferentially localizes at the cell membrane^29^ and specifically accumulates in mitochondria^30^. In the presence of molecular oxygen, HY can be activated by yellow light at 585 nm to generate cytotoxic ROS, inducing cell killing. These events activate the inflammatory responses and induce apoptosis. The detail reaction mechanism is shown in Fig. 8.

The inflammatory responses subsequently trigger the secretion of TNF-α, IL-6 and IFN-γ, which facilitate long-lasting immunity and the suppression of HUVECs growth. Additionally, HY-PDT also induces HUVEC cell apoptosis *via* mitochondrial apoptotic pathway. The activation of Bax, the suppression of Bcl-2 results in mitochondrial dysfunction. Furthermore, the increased of mitochondrial membrane permeability, accompanied with the release of cyto c, further activates caspase-9 and the downstream effector caspase-3, and eventually cause the PARP cleavage. Finally, HY-PDT could inhibit HUVEC growth by preventing the activation of the the VEGF-A-mediated PI3K/Akt pathway, which is initiated by suppressing VEGF-A expression. This, in turn, down-regulates the levels of the total Akt and p-Akt proteins to inhibit Akt activation, and stimulates the dephosphorylation of Bad, leading to the release of cyto c. On the other hand, the suppression of p-Akt may also activate downstream caspases; and ultimately cellular apoptosis occurs. Together, HY-PDT induces apoptosis *via* activating the mitochondrial-mediated caspase pathway and inhibiting the VEGF-A-mediated PI3K/Akt pathway activation. In conclusion, our studies suggested that HY-PDT can trigger cell death through a cascade of events including direct cell killing, activation of the inflammatory responses and induction of apoptosis.

## Discussion

In this study, HUVECs were used to explore the photocytotoxic effects of hypericin (HY). Our results showed HY had a very potent inhibiting effect on HUVECs at very low concentrations. Meanwhile, the mechanism of the HY-mediated photocytotoxicity was proposed as followed: First, HY is activated by yellow light at 585 nm, which has a strong penetrating ability for efficient absorption by HY^15^,^31^, in the presence of molecular oxygen, and cytotoxic ROS was generated. It kills the cells and triggers an inflammatory response, leading to the rapid secretion of inflammatory mediators, such as TNF-α, IL-6 and IFN-γ, and particularly IL-6. Subsequently, the secondary inflammatory mediators may recruit DCs, macrophages and neutrophils^32^; this events facilitate long-lasting immune suppression of HUVEC growth. Additionally, photoactivated HY-induced ROS could also inhibit cell growth by inducting apoptosis by activating a mitochondrial caspase pathway and preventing the activation of the VEGF-A-mediated PI3K/Akt pathway.

Our studies indicated that HY-PDT exhibited concentration-dependent photocytotoxic effect. As shown in Fig. 1b, both HY and haematoporphyrin (HP) showed the inhibitory effect on HUVECs; cell viability following the 2 μM HP-PDT was 81.0%, whereas that following the 0.125 μM HY -PDT was 38.5%. The IC_50_ value of HY was 0.067 μM, which was much less than that of HP (2 μM). It is worth noting that in the cell viability studies of HUVECs, no inhibition was found in Non-PDT groups, indicating that the inhibitory effect of HY or HP is light-dependent. HY displays negligible biological activity in the absence of light^33^.

Our studies revealed that the photocytotoxic effects of HY are resulted from the induced apoptosis, as seen from DAPI staining experiments (Fig. 2 and Supplementary Fig. 2S). We have observed the nuclear morphology of the treated cells changed in different extents, including chromatin condensation, marginalization and even DNA fragmentation, all of which are characteristics of apoptosis. In particular, when treated with 0.125 μM HY, the nuclei were collapsed, and the DNA was fragmented (Supplementary Fig. S2a). This effect was further validated by flow cytometry. It was also observed that HY- or HP-PDT-treated cells displayed phosphatidylserine exposure (Annexin V^+^/PI^–^), a hallmark of early apoptosis. Furthermore, the number of late apoptotic or necrotic cells (Annexin V^+^/PI^+^), which are characterized by plasma membrane damage, significantly increased. This explained why DNA fragmentation was only observed with the highest concentration of HY (0.125 μM). Additionally, excess ROS was detected, which is associated with apoptosis, as ROS promotes mitochondrial toxicity and macromolecular membrane damage, and consequently DNA fragmentation^19^,^34^,^35^. Thus, it is evident, that the excess ROS in mitochondria contribute to the mitochondrial caspase apoptotic signalling pathway^19^,^36^,^37^. Hence, we concluded that HY-PDT induces apoptosis by facilitating ROS production.

Importantly, our findings also revealed that the treatment with HY-PDT significantly increased the production of the inflammatory factors, including TNF-α and IL-6 (Fig. 3). These inflammatory factors are primarily modulated *via* intracellular ROS^38^,^39^. TNF-a, IFN-γ and IL-6 have been shown to be important in the promotion of an efficient innate immune response. Thus, our findings showing a remarkable increase in TNF-α, IL-6 and IFN-γ expression is consistent with a pro-inflammatory state involving the up-regulation of the innate immune system. Notably, the expression of the IL-6 mRNA and protein was more significantly up-regulated. IL-6 provokes specific cellular immune responses to inflammatory stimuli and also activates thymic lymphocytes (T-cells)^40^. However, HY-PDT did not affect the production of IL-1β or the immunosuppressive factors IL-10, suggesting that HY-PDT could promote the secretion of inflammatory mediators in a cell-selective manner. Our studies reveal that the HY-PDT can activate cell death *via* activation of the inflammatory response, sequentially inducing TNF-a, IFN-γ and IL-6 secretion. Such a cascade event thereby suggest that an additional potential of HY-PDT to suppress cell growth over the long term.

Previous studies have revealed that the photodynamic action of HY primarily targeted the mitochondria^41^. Rapid depletion of the ΔΨ_m_ leads to changes in mitochondrial membrane permeability, which is regulated by the balance of the pro- and anti-apoptotic Bcl-2 family proteins^42^. Thus, an increased ratio of pro- and anti-apoptotic protein expression, namely, the Bax/Bcl-2 ratio, may lead to the loss of mitochondrial membrane potential, resulting in the release of cyto c. Subsequently, the downstream effectors, caspase-9 and caspase-3 are activated, and PARP is cleaved. Eventually apoptosis occurs. In this studies, we did observed all these events. Importantly, we have observed the pan-caspase inhibitor (zVAD-fmk) or the specific Bax inhibitor (BIP) can reduce the effects on above-mentioned apoptosis-associated factors. These results indicate that HY-PDT induces apoptosis undergoing a mitochondrial-mediated pathway.

Interestingly, we found that down-regulated VEGF-A, Akt and p-Akt proteins and up-regulated dephosphorylated Bad (Fig. 7). Furthermore, this could be rescued by the addition of VEGF-A (Supplementary Fig. 7S). Based on these results, we concluded that HY-PDT could inhibit HUVEC growth by preventing the activation of the VEGF-A-induced PI3K/Akt pathway, which was initiated by the suppression of VEGF-A expression and subsequent down-regulation of the levels of the total Akt and p-Akt proteins, and eventually stimulating the Bad dephosphorylation^43^. Bad is a critical pro-apoptotic protein of the Bcl-2 family of proteins and is reportedly able to promote the release of cyto c to activate caspase-dependent apoptosis^44^. Putting all the data together, we concluded that HY-PDT could induce apoptosis by activating the mitochondrial caspase pathway and by inhibiting the VEGF-A-mediated PI3K/Akt pathway. These are alternative mechanisms to inhibit cell growth. However, PDT is an oxygen-consuming modality. In an adaptive response to the hypoxic stress, the cells might trigger angiogenesis by immediate up-regulating pro-angiogenic factors, such as VEGF^45^. Our observation of decreased VEGF-A expression at 24 h following PDT can likely be explained that reoxygenation occurred after 24 h following the HY-PDT treatment^46^.

In conclusion, our study demonstrated that HY can inhibit the cell growth and induce apoptosis in HUVECs; HY also exhibits more potent effects than HP in the PDT. Our studies further discovered a novel mechanism of cell death underlie the role of HY. HY can promote the production of cytotoxic ROS, leading to cell death and the activation of the inflammatory responses. Furthermore, HY can also induce apoptosis by activating the mitochondrial caspase pathway and inhibiting the activation of the VEGF-A-mediated PI3K/Akt pathway. Taken together, HY should represent an excellent alternative to HP in PWSs treatment, owing to its shorter half-life yet higher photocytotoxic effects at lower concentrations.

## Additional Information

**How to cite this article**: Zhang, Q. *et al.* Hypericin-photodynamic therapy induces human umbilical vein endothelial cell apoptosis. *Sci. Rep.* **5**, 18398; doi: 10.1038/srep18398 (2015).

## Supplementary Material

Supplementary Information

## Figures and Tables

**Table 1:** Primers used for quantitative real-time PCR.

Template	Forward primer (5′-3′)	Reverse primer (5′-3′)
TNF-α	TCAGTCAGTGGCCCAGAAGAC	GATACCCCTCACACTCCCCAT
IL-6	CGAGCCCACCGGGAACGAAA	GGACCGAAGGCGCTTGTGGAG
Bax	AGTGGCAGCTGACATGTTTT	GGAGGAAGTCCAATGTCCAG
Bcl-2	GCCCTGTGGATGACTGAGTA	GGCCGTACAGTTCCACAAAG
Cytochrome c	AGTGTTCCCAGTGCCACACCG	TCCTCTCCCCAGAATGATGCCTTT
Caspase-3	TGTGAGGCGGTTGTGGAAGAGT	AATGGGGGAAGAGGCAGGTGCA
Bad	CCTCAGGCCTATGCAAAAAG	AAACCCAAAACTTCCGATGG
GAPDH	CGGAGTCAACGGATTTGGTCGTAT	AGCCTTCTCCATGGTGGTGAAGAC

## References

[b1] Hackett C. B. & Langtry J. A. Basal Cell Carcinoma of the Ala Nasi Arising in a Port Wine Stain Treated Using Mohs Micrographic Surgery and Local Flap Reconstruction. Dermatologic Surgery 40, 590–592 (2014). 24456324 10.1111/dsu.12442

[b2] Jacobs A. H. & Walton R. G. The incidence of birthmarks in the neonate. Pediatrics 58, 218–222 (1976). 951136

[b3] Barsky S. H., Rosen S., Geer D. E. & Noe J. M. The nature and evolution of port wine stains: a computer-assisted study. Journal of Investigative Dermatology 74, 154–157 (1980). 7359006 10.1111/1523-1747.ep12535052

[b4] GERONEMUS R. G. & Ashinoff R. The Medical Necessity of Evaluation and Treatment of Port‐Wine Stains. The Journal of dermatologic surgery and oncology 17, 76–79 (1991). 1991884 10.1111/j.1524-4725.1991.tb01597.x

[b5] Strauss R. P. & Resnick S. D. Pulsed dye laser therapy for port-wine stains in children: psychosocial and ethical issues. The Journal of pediatrics 122, 505–510 (1993). 8463892 10.1016/s0022-3476(05)83527-9

[b6] Wagner K. D. & Wagner R. Jr The necessity for treatment of childhood port-wine stains. Cutis 45, 317–318 (1990). 2192830

[b7] Yuan K.-H., Li Q., Yu W.-L. & Huang Z. Photodynamic therapy in treatment of port wine stain birthmarks—recent progress. Photodiagnosis and photodynamic therapy 6, 189–194 (2009). 19932450 10.1016/j.pdpdt.2009.08.001

[b8] Yuan K.-H. *et al.* Comparison of photodynamic therapy and pulsed dye laser in patients with port wine stain birthmarks: a retrospective analysis. Photodiagnosis and photodynamic therapy 5, 50–57 (2008). 19356637 10.1016/j.pdpdt.2007.12.001

[b9] Xiao-Xi L., Wei W., Shuo-fan W., Chuan Y. & Ti-Sheng C. Treatment of capillary vascular malformation (port-wine stains) with photochemotherapy. Plastic and reconstructive surgery 99, 1826–1830 (1997). 9180705

[b10] Bellnier D. A. *et al.* Clinical Pharmacokinetics of the PDT Photosensitizers Porfimer Sodium (Photofrin), 2‐[1‐Hexyloxyethyl]‐2‐Devinyl Pyropheophorbide‐a (Photochlor) and 5‐ALA‐Induced Protoporphyrin IX. Lasers in surgery and medicine 38, 439–444 (2006). 16634075 10.1002/lsm.20340

[b11] Zalar G. L., Poh-Fitzpatrick M., Krohn D. L., Jacobs R. & Harber L. C. Induction of drug photosensitization in man after parenteral exposure to hematoporphyrin. Archives of dermatology 113, 1392–1397 (1977). 911167

[b12] Qin Z.-P., Li K.-L., Ren L. & Liu X.-J. Photodynamic therapy of port wine stains—a report of 238 cases. Photodiagnosis and photodynamic therapy 4, 53–59 (2007). 25047192 10.1016/j.pdpdt.2007.01.001

[b13] Qiu H., Gu Y., Wang Y. & Huang N. Twenty Years of Clinical Experience with a New Modality of Vascular‐Targeted Photodynamic Therapy for Port Wine Stains. Dermatologic Surgery 37, 1603–1610 (2011). 21883647 10.1111/j.1524-4725.2011.02129.x

[b14] Gu Y., Huang N., Liang J., Pan Y. & Liu F. Clinical study of 1949 cases of port wine stains treated with vascular photodynamic therapy (Gu’s PDT). Annales de dermatologie et de vénéréologie 134, 241–244 (2007). 17389848 10.1016/s0151-9638(07)91816-5

[b15] Luksiene Z. & de Witte P. Hypericin as novel and promising photodynamic therapy tool: studies on intracellular accumulation capacity and growth inhibition efficiency. Medicina (Kaunas, Lithuania) 39, 677–682 (2002). 12878823

[b16] Redmond R. W. & Gamlin J. N. A compilation of singlet oxygen yields from biologically relevant molecules. Photochemistry and photobiology 70, 391–475 (1999). 10546544

[b17] Ritz R. *et al.* Photodynamic therapy of malignant glioma with hypericin: comprehensive *in vitro* study in human glioblastoma cell lines. International journal of oncology 30, 659–668 (2007). 17273767

[b18] Garg A. D. & Agostinis P. ER stress, autophagy and immunogenic cell death in photodynamic therapy-induced anti-cancer immune responses. Photochemical & Photobiological Sciences 13, 474–487 (2014). 24493131 10.1039/c3pp50333j

[b19] Barathan M. *et al.* Hypericin-photodynamic therapy leads to interleukin-6 secretion by HepG2 cells and their apoptosis via recruitment of BH3 interacting-domain death agonist and caspases. Cell death & disease 4, e697 (2013). 23807226 10.1038/cddis.2013.219 PMC3702308

[b20] Garg A. D. *et al.* ROS-induced autophagy in cancer cells assists in evasion from determinants of immunogenic cell death. Autophagy 9, 1292–1307 (2013). 23800749 10.4161/auto.25399

[b21] Krammer B. & Verwanger T. Molecular response to hypericin-induced photodamage. Current medicinal chemistry 19, 793–798 (2012). 22214453 10.2174/092986712799034842

[b22] Dewaele M. *et al.* Autophagy pathways activated in response to PDT contribute to cell resistance against ROS damage. Journal of cellular and molecular medicine 15, 1402–1414 (2011). 20626525 10.1111/j.1582-4934.2010.01118.x PMC4373339

[b23] Quiney C., Billard C., Mirshahi P., Fourneron J. & Kolb J. Hyperforin inhibits MMP-9 secretion by B-CLL cells and microtubule formation by endothelial cells. Leukemia 20, 583–589 (2006). 16467866 10.1038/sj.leu.2404134

[b24] Li Z. h. *et al.* Hypericin Damages the Ectatic Capillaries in a Roman Cockscomb Model and Inhibits the Growth of Human Endothelial Cells More Potently Than Hematoporphyrin Does through Induction of Apoptosis. Photochemistry and photobiology 90, 1368–1375 (2014). 25065502 10.1111/php.12323

[b25] Brockmöller J. *et al.* Hypericin and pseudohypericin: pharmacokinetics and effects on photosensitivity in humans. Pharmacopsychiatry 30, 94–101 (1997). 9342768 10.1055/s-2007-979527

[b26] Fulda S. Modulation of mitochondrial apoptosis by PI3K inhibitors. Mitochondrion 13, 195–198 (2013). 22580303 10.1016/j.mito.2012.05.001

[b27] Zhou J., Gan N., Zhang W., Lu W. & Xie X. Proliferation suppression and apoptosis of ovarian carcinoma cells induced by small interfering RNA against vascular endothelial growth factor. Journal of Obstetrics and Gynaecology Research 36, 232–238 (2010). 20492371 10.1111/j.1447-0756.2010.01196.x

[b28] Zhou H. B. *et al.* Suppression of vascular endothelial growth factor via siRNA interference modulates the biological behavior of human nasopharyngeal carcinoma cells. Japanese journal of radiology 29, 615–622 (2011). 21956366 10.1007/s11604-011-0603-9

[b29] Crnolatac I. *et al.* *In vitro* accumulation and permeation of hypericin and lipophilic analogues in 2-D and 3-D cellular systems. International journal of oncology 30, 319–324 (2007). 17203212

[b30] Davids L. M., Kleemann B., Kacerovská D., Pizinger K. & Kidson S. H. Hypericin phototoxicity induces different modes of cell death in melanoma and human skin cells. Journal of Photochemistry and Photobiology B: Biology 91, 67–76 (2008). 10.1016/j.jphotobiol.2008.01.011 18342534

[b31] Uzdensky A. B. *et al.* Photodynamic effect of hypericin and a water-soluble derivative on isolated crayfish neuron and surrounding glial cells. Journal of Photochemistry and Photobiology B: Biology 72, 27–33 (2003). 10.1016/j.jphotobiol.2003.08.008 14644563

[b32] Ritz R. *et al.* Subcellular colocalization of hypericin with respect to endoplasmic reticulum and Golgi apparatus in glioblastoma cells. Anticancer research 28, 2033–2038 (2008). 18751371

[b33] Kim Y.-W., Bae S. M., Battogtokh G., Bang H. J. & Ahn W. S. Synergistic anti-tumor effects of combination of photodynamic therapy and arsenic compound in cervical cancer cells: *in vivo* and *in vitro* studies. PloS one 7, e38583 (2012). 22715393 10.1371/journal.pone.0038583 PMC3371011

[b34] Bulina M. E. *et al.* A genetically encoded photosensitizer. Nature biotechnology 24, 95–99 (2005). 10.1038/nbt1175 16369538

[b35] Butler M. C., Itotia P. N. & Sullivan J. M. A high-throughput biophotonics instrument to screen for novel ocular photosensitizing therapeutic agents. Investigative ophthalmology & visual science 51, 2705–2720 (2010). 19834043 10.1167/iovs.08-2862 PMC2868480

[b36] Lee M. K., Lu Y., Di L. Q. & Xu H. Q. Protection of Tong-Sai-Mai Decoction against Apoptosis Induced by H2O2 in PC12 Cells: Mechanisms via Bcl-2-Mitochondria-ROS-INOS Pathway. Evidence-based complementary and alternative medicine. (2014) [published online 10.1155/2014/371419]. PMC4227446 25404948

[b37] Turrens J. F. Mitochondrial formation of reactive oxygen species. The Journal of physiology 552, 335–344 (2003). 14561818 10.1113/jphysiol.2003.049478 PMC2343396

[b38] Cerutti P. & Trump B. F. Inflammation and oxidative stress in carcinogenesis. Cancer cells (Cold Spring Harbor, NY: 1989) 3, 1–7 (1991). 2025490

[b39] Johar D. *et al.* Inflammatory response, reactive oxygen species, programmed (necrotic-like and apoptotic) cell death and cancer. Roczniki Akademii Medycznej w Bialymstoku (1995) 49, 31–39 (2003). 15631311

[b40] Kick G., Messer G., Goetz A., Plewig G. & Kind P. Photodynamic therapy induces expression of interleukin 6 by activation of AP-1 but not NF-κB DNA binding. Cancer research 55, 2373–2379 (1995). 7757989

[b41] Theodossiou T. A., Hothersall J. S., De Witte P. A., Pantos A. & Agostinis P. The multifaceted photocytotoxic profile of hypericin. Molecular pharmaceutics 6, 1775–1789 (2009). 19739671 10.1021/mp900166q

[b42] Bouillet P. & Strasser A. BH3-only proteins—evolutionarily conserved proapoptotic Bcl-2 family members essential for initiating programmed cell death. Journal of cell science 115, 1567–1574 (2002). 11950875 10.1242/jcs.115.8.1567

[b43] Datta S. R. *et al.* Akt phosphorylation of BAD couples survival signals to the cell-intrinsic death machinery. Cell 91, 231–241 (1997). 9346240 10.1016/s0092-8674(00)80405-5

[b44] Jiang P., Du W., Heese K. & Wu M. The Bad guy cooperates with good cop p53: Bad is transcriptionally up-regulated by p53 and forms a Bad/p53 complex at the mitochondria to induce apoptosis. Molecular and cellular biology 26, 9071–9082 (2006). 17000778 10.1128/MCB.01025-06 PMC1636833

[b45] Pugh C. W. & Ratcliffe P. J. Regulation of angiogenesis by hypoxia: role of the HIF system. Nature medicine 9, 677–684 (2003). 10.1038/nm0603-677 12778166

[b46] Uehara M., Inokuchi T., Sano K. & ZuoLin W. Expression of vascular endothelial growth factor in mouse tumours subjected to photodynamic therapy. European Journal of Cancer 37, 2111–2115 (2001). 11597392 10.1016/s0959-8049(01)00243-x

