# Supplementary material for: Hypericin-photodynamic therapy induces human umbilical vein endothelial cell apoptosis
Source: Sci Rep. 2015 Dec 17;5:18398. doi: 10.1038/srep18398 (PMC4682094; doi:10.1038/srep18398)

Supplementary Information

Hypericin-photodynamic therapy induces human umbilical vein endothelial cell apoptosis

Qian Zhang[1], Zhuo-heng Li[1], Yuan-yuan Li[1], San-jun Shi[1], Shi-wen Zhou[2], Yuan-yuan Fu[1], Qing Zhang[1], Xue Yang[1], Ruo-qiu Fu[1], and Lai-chun Lu[1*]

[1]*Department of Pharmacy, Institute of Surgery Research, Daping Hospital, Third Military Medical University, Chongqing, 400042, P. R. China*

[2]*Office of Clinical Pharmacological Center, Xinqiao Hospital, Third Military Medical University, Chongqing, 400037, P. R. China*

Correspondence and requests for materials should be addressed to L.L. (lulab2014@126.com)

Supplementary Figure Legends

Supplementary Figure S1. Cell viability, procaspase-3 and cleaved PARP proteins expression in HUVECs at 0, 1, 6, 12 and 24 h after exposure to laser light. HUVECs were treated with increasing concentrations of HY (0.031, 0.062 and 0.125 μM) for 24 h and were then exposed to a 585-nm LED light at a dose of 1.0 J/cm^2. Subsequently, the cells were further incubated for 0, 1, 6, 12 or 24 h, in the dark. (a) The inhibitory effect of HY-PDT on the growth of HUVECs was examined using a MTT assay. Viability of the treated cells was compared with that of cells at 0h post-PDT (100%). Data are presented as means ±S.D. (n = 3); *n.s.*, $P > 0.05$, ***$P < 0.001$, compared with 0 h. (b) The expression of procaspase-3 and cleaved PARP in HUVECs treated with HY (0.062 and 0.125 μM) was determined by western blotting. Densitometric values of procaspase-3 and cleaved PARP were analysed using AlphaEaseFC 4.0 software. Protein expression was normalized to that at 0h (100%). Data are presented as means ±S.D. (n = 3), *n.s.*, $P > 0.05$, **$P < 0.01$, ***$P < 0.001$, compared to 0 h.

Supplementary Figure S2. HY-PDT induces apoptosis in HUVECs. HUVECs were treated with increasing concentrations of HY (0.031, 0.062 and 0.125 μM) and HP (2 μM) for 24 h and were then exposed to a 585-nm LED light at a dose of 1.0 J/cm^2. Subsequently, cells were further incubated for an additional 24 h. The cells were then harvested. (a) HUVECs were stained with DAPI to visualize cell nuclei and were observed under fluorescence microscopy. Images are representative of three independent experiments. White arrows indicate condensed chromatin, marginalization or nuclei that had fragmented into apoptotic bodies. Bars, 100 μm. (b) Representative images of AnnexinV$^+$PI$^+$ flow cytometry. (c) Quantification of the number of AnnexinV$^+$PI$^+$ cells. Data are presented as means ±S.D. (n = 3); *$P < 0.05$, ***$P < 0.001$, compared to vehicle control, $^{\#}P < 0.05$, $^{\#\#\#}P < 0.001$, compared to HP.

Supplementary Figure S5. HY-PDT induces ΔΨm collapse in HUVECs. HUVECs were treated with increasing concentrations of HY (0.031, 0.062 and 0.125 μM) and HP (2 μM) for 24 h and were then exposed to a 585-nm LED light at a dose of 1.0 J/cm^2. Subsequently, cells were incubated for an additional 24 h. The ΔΨm was reduced following HY- or HP-PDT, as determined using a JC-1 probe. CCCP, which could clearly cause the depletion of ΔΨm, was used as a positive control. The ratio of red and green fluorescence intensities represents the ΔΨm of HUVECs. Following PDT treatment with increasing doses of HY, red fluorescence became weaker and green fluorescence became stronger, as observed under fluorescence microscopy. (a) Images detected under fluorescence microscopy are representative of three independent experiments. Bars, 100 μm. (b) Bar graphs show the ratio of red and green fluorescence

intensities from JC-1, as detected under fluorescence microscopy. The ratio of red and green fluorescence intensities was normalized to that of the vehicle control (100%). Data are presented as means ±S.D. (n = 3); ***$P < 0.001$, compared to the vehicle control, $^{\#}P < 0.05$, $^{\#\#\#}P < 0.001$, compared to HP.

Supplementary Figure S6. HY-PDT induces the expression of apoptotic mediators in HUVECs. HUVECs were treated with increasing concentrations of HY (0.031, 0.062 and 0.125 μM) and HP (2 μM) for 24 h and were then exposed to a 585-nm LED light at a dose of 1.0 J/cm^2. Subsequently, cells were incubated for an additional 24 h. Total protein and RNA were extracted from PDT-treated cells, and western blot analysis and two-step qRT-PCR reactions were performed, respectively. (a) Western blot analysis of Bcl-2, Bax, cleaved caspase-9, procaspase-3 and cleaved PARP in HUVECs treated with HY- or HP-PDT. (b and c) Densitometric measurements of cleaved caspase-9, procaspase-3 and cleaved PARP were analysed using AlphaEaseFC 4.0 software. Protein expression was normalized to those of the vehicle control (100%). Data are presented as means ±S.D (n = 3); *$P < 0.05$, **$P < 0.01$, ***$P < 0.001$, compared to vehicle control cells, $^{\#}P < 0.05$, $^{\#\#}P < 0.01$, compared with HP. (d, e and f) Levels of mRNA of Bax/Bcl-2, cyto c and caspase-3 were examined by qRT-PCR. The gene expression levels were normalized to those of GAPDH and were calibrated to vehicle controls. Data are presented as means ±S.D. (n = 3); *$P < 0.05$, **$P < 0.01$, ***$P < 0.001$, compared to vehicle control, $^{\#\#}P < 0.01$, $^{\#\#\#}P < 0.001$, compared to HP.

Supplementary Figure S7. HY-PDT inhibits VEGF-A-mediated PI3K/Akt pathway activation in HUVECs. HUVECs were treated with increasing concentrations of HY (0.031, 0.062 and 0.125 μM) and HP (2 μM) for 24 h and were then exposed to a 585-nm LED light at a dose of 1.0 J/cm^2. Subsequently, cells were incubated for an additional 24 h. Total protein and RNA were extracted from PDT-treated cells and western blot analysis and two-step qRT-PCR reactions were performed, respectively. (a) Western blot analysis of VEGF-A, p-Akt (Ser473), Akt and Bad in HUVECs treated with HY- or HP-PDT. (b, c and d) Densitometric values of VEGF-A, p-Akt/Akt and Bad were analysed using AlphaEaseFC 4.0 software. Protein expression levels were normalized to those of the vehicle control (100%). The data are presented as means ±S.D. (n = 3); *$P < 0.05$, **$P < 0.01$, ***$P < 0.001$, compared to the vehicle control, $^{\#}P < 0.05$, $^{\#\#}P < 0.01$, $^{\#\#\#}P < 0.001$, compared to HP. (e) Bad mRNA levels were assessed by qRT-PCR. The gene expression levels were normalized to those of GAPDH and were calibrated to vehicle controls. Data are presented as means ±S.D. (n = 3), **$P < 0.01$, ***$P < 0.001$, compared to the vehicle control, $^{\#}P < 0.05$, $^{\#\#}P < 0.01$, compared to HP.

Supplementary Figure S1

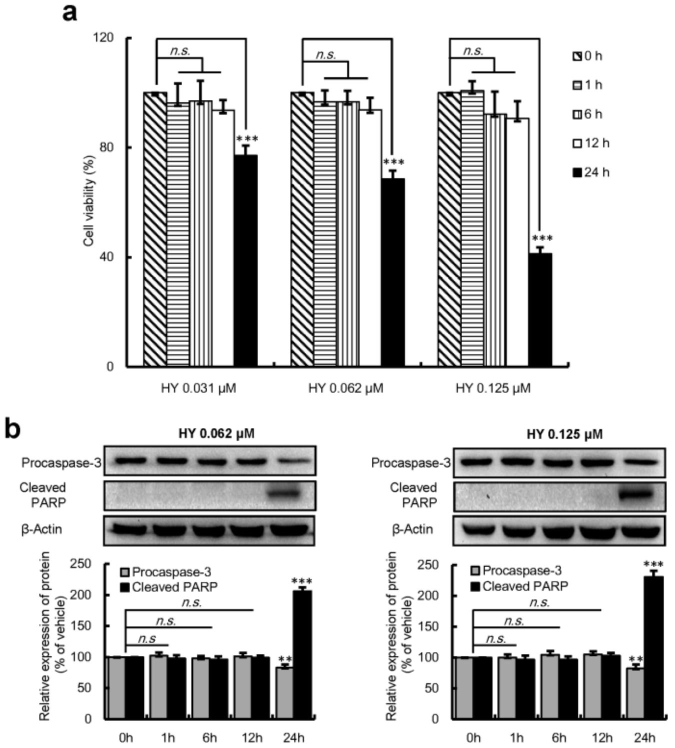

Supplementary Figure S2

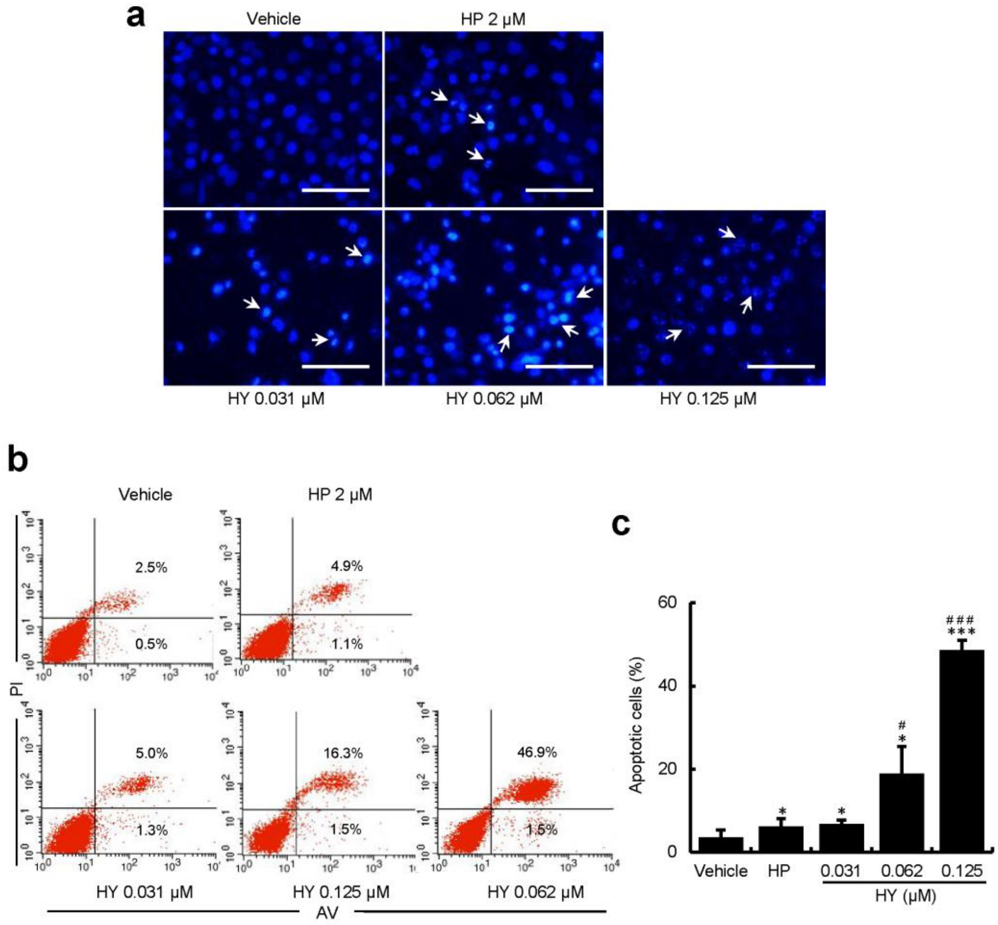

Supplementary Figure S5

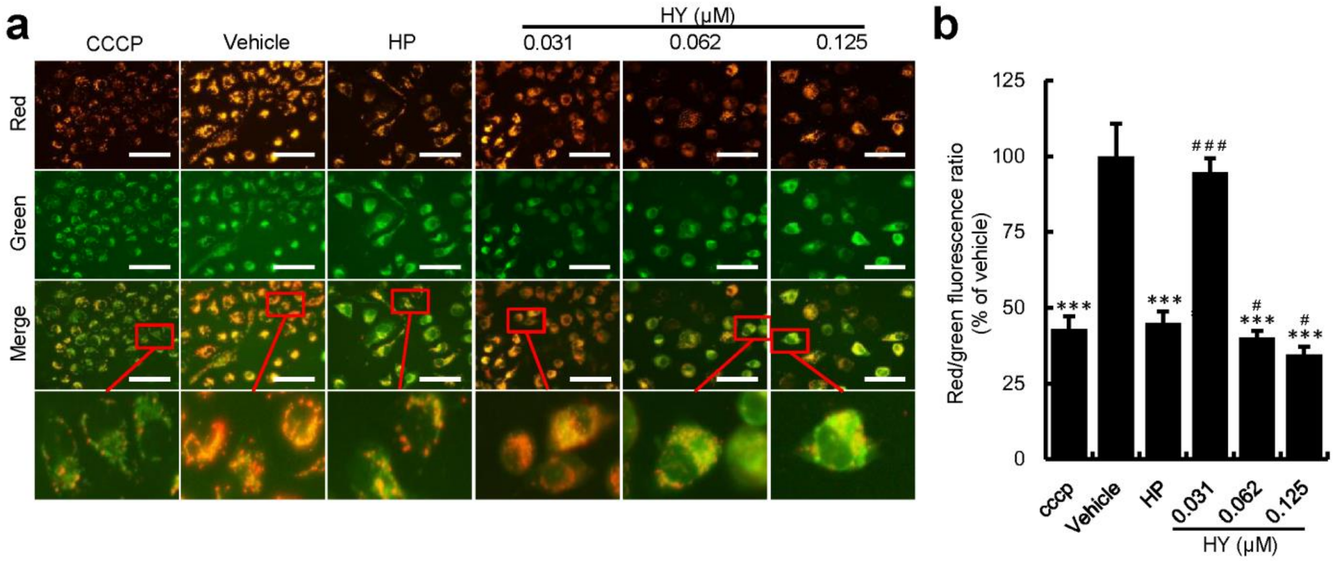

Supplementary Figure S6

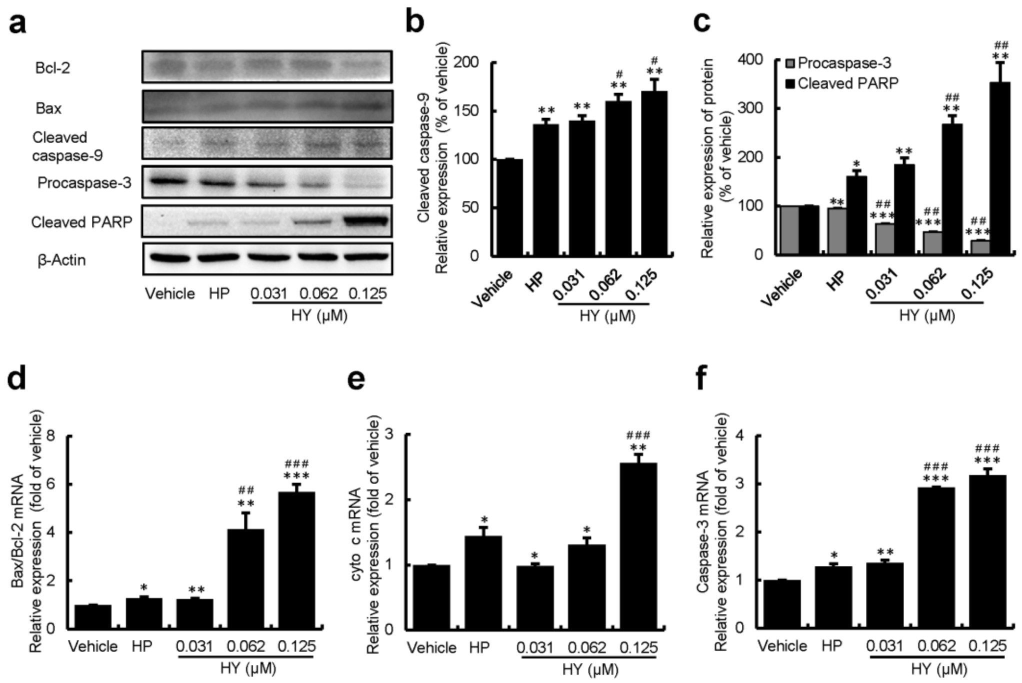

Supplementary Figure S7

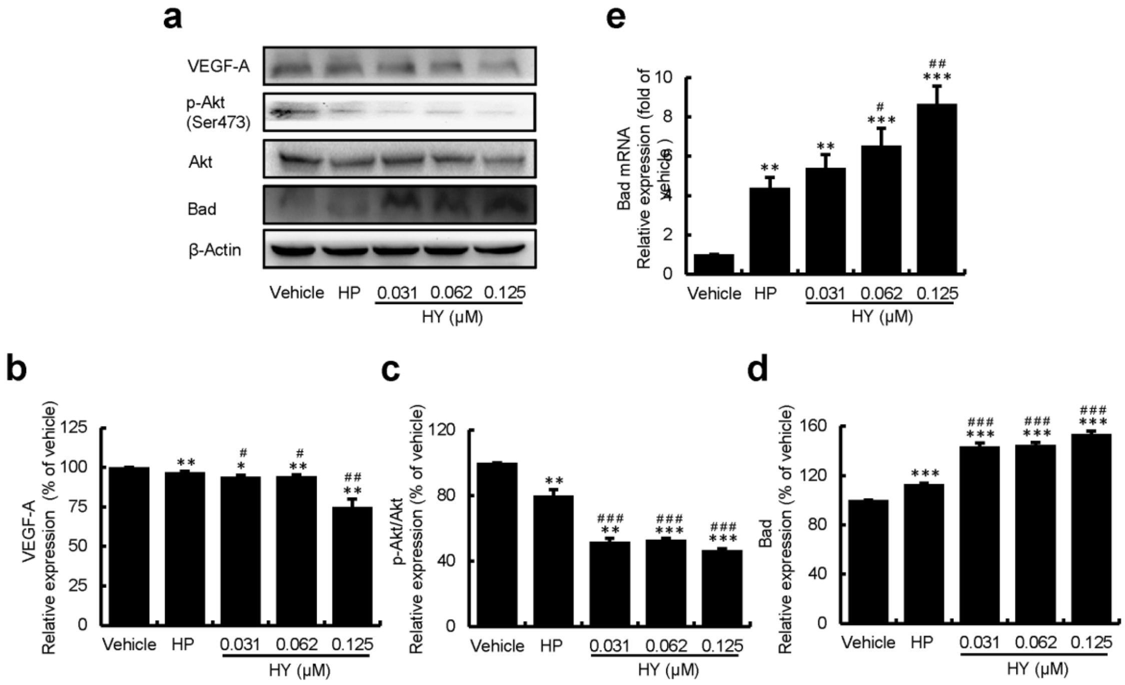

Supplement: Supplementary Information [file srep18398-s1.pdf]
